# Effects of single-use alfentanil versus propofol on cognitive functions after colonoscopy: A randomized controlled trial

**DOI:** 10.1016/j.heliyon.2023.e17061

**Published:** 2023-06-12

**Authors:** Xiwen Zhu, Xuehan Chen, Xuemei Zheng, Hongyao Lyu, Jie Chen, Ai Yan, Qi Liu, Shiqi Li, Yamei Zhang, Ting Wang, Guangyou Duan, He Huang

**Affiliations:** a Department of Anesthesiology, The Second Affiliated Hospital, Chongqing Medical University, Chongqing, China; b Department of Preventive Medicine, West China School of Public Health, Sichuan University, Chengdu, Sichuan, China; c Department of Psychology, The Second Affiliated Hospital, Chongqing Medical University, Chongqing, China

**Keywords:** Alfentanil, Propofol, Colonoscopy, Cognitive function

## Abstract

**Purpose:**

Colonoscopy is often accompanied by short-term postoperative cognitive decline. We aimed to explore whether single-use alfentanil for patients undergoing elective colonoscopy could reduce cognitive impairment at discharge compared with propofol.

**Patients and methods:**

172 adult patients undergoing elective colonoscopy were randomized to receive intravenous propofol at 2 mg/kg (group P) or alfentanil at 10 μg/kg (group A); 40 healthy volunteers were included in the blank group. Cognitive function was considered the primary outcome and was measured using five neuropsychological tests before sedation and discharge. The z-score method was used to determine cognitive dysfunction according to z-score >1.96 in two types of neuropsychological tests. Other outcomes included discharge time, vital signs, associated adverse events during colonoscopy, and the satisfaction level of patients and endoscopic physicians.

**Results:**

164 patients (78 in group A and 86 in group P) completed the study protocol. At discharge, the incidence of cognitive dysfunction in group P was 23% and was significantly lower in the alfentanil group (2.5%), with a relative risk of 0.11 (95% confidence interval: 0.03–0.46, P < 0.001). The incidence of hypotension in group A was lower than that in group P (3.8% vs 22.1%, relative risk = 0.17 [95% confidence interval: 0.05–0.46, P = 0.001]), and the discharge time in group A was shorter than that in group P (5 [(Rutter and et al., 2016; Zhang and et al., 2013; Hirsh and et al., 2006; Zhou and et al., 2021; Singh and et al., 2008; Ko and et al., 2010; Sargin et al., 2019) 3–93–9 vs 13 [(Ekmekci and et al., 2017; Eberl and et al., 2012; Eberl and et al., 2014; N'Kaoua and et al., 2002; Chung et al., 1995; Berger and et al., 2019; Quan and et al., 2019; Deng and et al., 2021; Gualtieri and Johnson, 2006) 10–1810–18 min, P < 0.001).

**Conclusion:**

For patients undergoing colonoscopy, single-use alfentanil causes less damage to postoperative cognitive function, less risk of hypotension, and shorter discharge time than propofol.

## 1 Introduction

Colonoscopy is the gold standard method for cancer screening and early detection of adenomas, which can effectively reduce the morbidity and mortality of colorectal cancer and ulcerative colitis [1,2]. It has become a routine diagnostic and treatment method, while tens of millions of people undergo this procedure every year [3,4]. Although most colonoscopies are performed under sedation, the choice of anesthetic may be influenced by national and cultural differences, the wishes of the patient, and the endoscopist's attitude toward the examination [5]. Propofol is currently the most used intravenous anesthetic during colonoscopies and is also widely used for routine outpatient endoscopies, with a utilization rate exceeding 60% [6]. Propofol can achieve a good sedative effect and has a rapid onset time [7],however, studies have suggested that propofol sedation can induce cardiovascular events, prolong hospital stay, and lead to cognitive impairment [8,9].

Rapid recovery of cognitive function is significant for a growing number of people who hope to be able to work or drive immediately after endoscopic procedures [10]. Therefore, exploring other means of anesthesia for colonoscopies is necessary to reduce the occurrence of postoperative cognitive dysfunction and the loss of working time.

Alfentanil is a short-acting μ-opioid analgesic that has a minor effect on the circulatory system and a low incidence of respiratory depression [11]. One study suggested that a single dose of alfentanil can be used for painless colonoscopic procedures, which can provide sufficient analgesia and mild sedation, and patients can meet the discharge criteria earlier [12]. In addition, one study suggested that alfentanil had little impact on memory within a short time [13]. However, there are currently no studies on cognitive function in patients undergoing colonoscopy with alfentanil alone. The aim of this study was to compare the cognitive effects of single-use alfentanil and propofol on two groups of patients undergoing colonoscopic procedures when they met traditional discharge criteria [14], as well as, discharge time, adverse events, and satisfaction, and to comprehensively explore the effectiveness and feasibility of alfentanil alone for colonoscopy.

## 2 Material and methods

### 2.1 Study design

A total of 172 outpatients undergoing colonoscopic procedures were randomized in a double-blind, randomized controlled trial to compare the cognitive effects of single-use alfentanil and propofol. The study was approved by the Medical Ethics Committee of the Second Affiliated Hospital of Chongqing Medical University (approval number: 2021-60) and registered at www.chictr.org.cn, China Clinical Trials Registration Center (registration ID: ChiCTR2100048639). Informed consent was obtained from all patients in this experiment.

### 2.2 Study population

172 outpatients undergoing colonoscopic procedures were recruited for this study. The control group (group C) consisted of 40 volunteers who, to exclude the learning effect, matched with the sex, age, and educational background of 172 patients. All patients were recruited from the appointment list of surgical colonoscopies at the Second Affiliated Hospital of Chongqing Medical University from July 12, 2021, to September 29, 2021. All patients followed a standardized protocol for the colonoscopy.

The inclusion criteria were age, 18–65 years; physiological status, American Society of Anesthesiologists classification I–II; a preoperative Mini-Mental State Exam (MMSE) score ≥24; requirement of colonoscopy with anesthesia; right-handedness; and no history of anesthesia drug allergy. Exclusion criteria included the presence of known mental diseases, the lack of communication and other cognitive abilities, inability to complete the preoperative evaluation (owing to color blindness, hand disability, and so on), abuse or application of other narcotic sedative analgesics, and long-term alcoholism, morbid obesity (body mass index >30 kg/m2), a history of anesthesia within 7 days before the procedure, allergy to study drugs or contraindications of propofol and alfentanil, and failure to follow the study protocol.

Family members who matched the age and educational background of patients were recruited for group C. The inclusion criteria were: volunteer age, 18–65 years; volunteer MMSE score ≥24; and right-handedness. The exclusion criteria are the same as test group.

### 2.3 Randomization and blinding

According to a simple randomization method, 172 envelopes were prepared in a 1:1 ratio and written into the alfentanil group (group A) and propofol group (group P). The patients were equally divided into groups A and P by randomly extracting the envelopes. Randomly selected results were maintained at the study site using the patient original data table until the end of the study. The anesthesiologist administered the corresponding anesthetic to the patient according to the random number in the envelope. The investigators of the neuropsychological tests (NPTs) were unaware of the subgroups of drugs administered.

### 2.4 Interventions

**Fig. 1:**
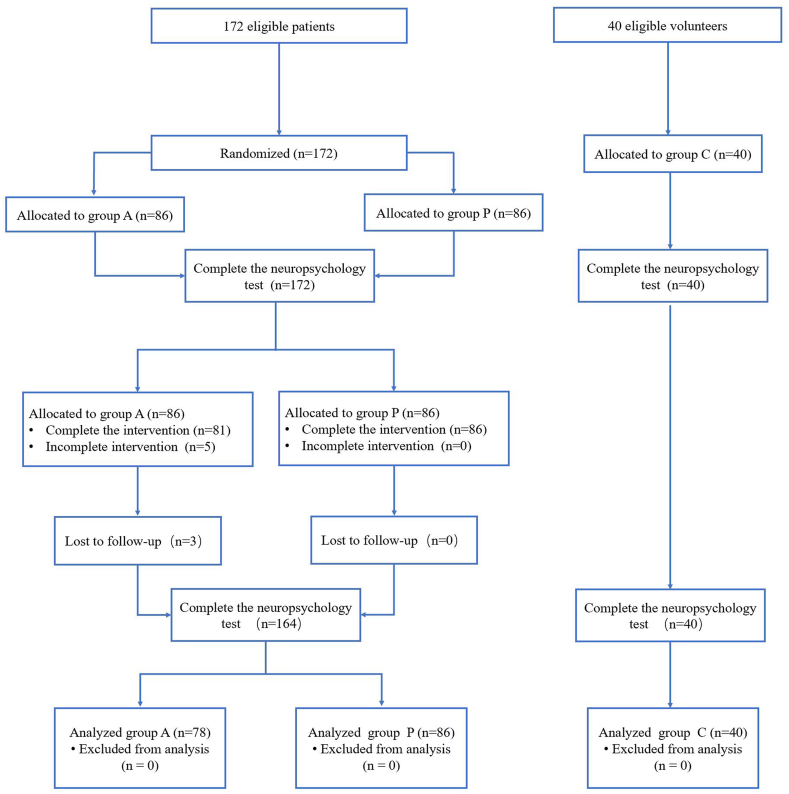
Consolidated Standards of Reporting Trials diagram.

All patients included in the study were examined by the same group of endoscopists, who had performed more than 1,000 endoscopies before participating in the study. An experienced anesthesiologist administered the anesthesia during the procedure. Demographic information was obtained preoperatively from their medical records and by questioning the patient. After the patient was brought into the endoscopy room, venous access was obtained and oxygen was provided through a nasal oxygen tube at 3 L/min, while noninvasive blood pressure, peripheral oxygen saturation, and heart rate were monitored (Fig. 1).

The patients in Group A were induced with 10 μg/kg alfentanil (Yichang Humanwell Pharmaceutical, Yichang, China) (diluted to 20 mL and pushed for 30 s) so that the sedation score Observer's Assessment of Alertness/Sedation Scale (OAA/S) ≤ 4. The OAA/S scores were measured every 3 min. If the patient could not tolerate colonoscopy because of pain, 5 μg/kg was added.

The patients in group P were administered with 2 mg/kg propofol (Xi’ an Libang Clinical Nutrition Corporation, Xi’ an, China). The target-controlled bolus injection of propofol resulted in an OAA/S ≤ 4, which was measured every 3 min. According to the patient's body motion response, 0.2–0.5 mg/kg of propofol could be added each time intravenously.

### 2.5 Primary outcome

The primary outcome of this study was the incidence of cognitive dysfunction, which was determined using five NPTs. The same professionally trained researcher assessed the whole process, from preoperative cognitive function (baseline) evaluation to discharge standards. Based on the area of clinical hypofunction exhibited and the NPTs recommended by the International Study Group of Postoperative Cognitive Dysfunction [[15], [16], [17]], the selected combinations of NPTs were the finger-tapping test, digital span test (DST), digital symbol substitution test (DSST), Stroop test, and Schulte grid test. All cognitive tasks were performed using intelligent electronic devices, except for DSST, which was tested with paper and pen. The cognitive ability of group C was assessed twice at 50-min intervals.

The finger tapping test is one of the most used tests in neuropsychology to evaluate the fine motor ability of human fingers [18]. Participants were asked to click in the required area as quickly as possible, and the tablet automatically recorded the completion time. The participants were asked to complete 50 tests with each hand and take a short rest between each test; only the fastest time for each hand was recorded.

The Schult table is used to evaluate the attention level of patients [19,20]. With 16 grids in the center of the screen that was randomly filled with Arabic numerals 1–16. Participants were asked to click on digital positions with their hands in the order of 1–16; the following digital click can be performed only after clicking on the correct position. The test was repeated twice for each formal test, and the score was recorded as the shortest time.

The DST [21]was performed to assess working memory and attention span by asking patients to immediately recall and input the correct numbers into the electronic device after seeing randomly occurring digit strings and by increasing the number string length by one digit at a time after the memory was correct. The number string length that was eventually recalled was recorded.

The DSST [16]is a good indicator for testing the “liquid intelligence, " such as the attention and executive abilities and is very sensitive to extensive brain dysfunction. The test required the patients to transcribe numbers into symbols in the line of sight quickly. The score was the number of correct symbols recorded within 20 s.

The Stroop color-word test evaluates distraction and conditioned suppression by asking patients to recognize the meaning of words quickly [22]. Words with different colors and definitions but the same meaning (such as “green” words written in red) appeared in the middle of the screen. The participants were asked to quickly select the definition of the word. The completion time was recorded by converting error numbers to times in the total completion time.

### 2.6 Other outcomes

The patient's vital signs (pulse oxygen saturation, heart rate, and noninvasive blood pressure) and OAA/S scores were recorded every 3 min during the colonoscopy. Respiratory depression (SpO2 < 90%), hypotension (blood pressure decreased by 20%), bradycardia (<50 beats/min), nausea, and emesis were recorded as adverse reactions. After the procedure, the maximum Numeric Rating Scale (NRS) pain score [23]was recorded.

The time from insertion of the endoscope to the end of its removal was recorded as the operation time. The discharge time was calculated as the time from endoscope removal until the patient reached the discharge standard. At the end of the procedure, the satisfaction of the endoscopist with the anesthesia effect and the satisfaction of the patient with the treatment process were assessed according to the 5- point Likert scale [22].

### 2.7 Sample size

The incidence of cognitive decline was the primary outcome of the study. In a preliminary study, we found that the incidence of cognitive decline was 22% (4/18) with propofol, while no cognitive decline was observed in the alfentanil group. We assumed that the incidence of cognitive decline in the alfentanil group was ≤5%. Based on a significance level of 0.05 and a power of 0.9, 77 patients in each group were required. Considering the missing follow-up rate of approximately 10%, the total required sample size was 172 patients, with 86 patients in each group.

### 2.8 Statistical analysis

Continuous data were subjected to a normality test. The mean value (SD) was used to summarize if the data were normally distributed, and the median value (interquartile range [IQR]) was used to summarize if the data were not normally distributed. In this study the primary aim is to compare the difference of cognitive dysfunction between Alfentanil group and Propofol group. Normally distributed data between the two groups were compared using an independent *t*-test. Comparisons of skewed data were performed using the Mann–Whitney *U* test. Categorical data are summarized as numbers (%) and were compared using the chi-square test or Fisher's exact test. The relative risk with a 95% confidence interval was calculated for the incidence of cognitive dysfunction in the two groups. All data were analyzed using SPSS software (IBM SPSS Statistics for Windows, Version 20.0. Armonk, NY: IBM Corp.). All reported *P*-values were bilateral and two-sided, with P < 0.05 indicating statistically significant difference.

We analyzed the decline in cognitive function using the z-score method. According to the mean and standard deviation of the scores before and after each test in the control group, the Z score of the corresponding test in the experimental group was calculated. By definition, Z score >1.96 on two or more tests in our neuropsychological test combination is the standard for cognitive impairment [24,25]. The z-score is a relative quantity that represents the distance between the measured value and the mean (without units) and is equal to the difference between the measured value x and the mean, μ, divided by the standard deviation, σ, in the following equation:

Z=(x－μ)÷ σ。

## 3 Results

172 patients were enrolled in the study and randomly divided into group P and group A. In Group A, 5 patients were excluded because of changing anesthesia methods, and three refused to complete the follow-up NPTs. Finally, 78 patients in group A and 86 patients in group P were included in the statistical analysis. 40 volunteers completed two cognitive function tests. The demographic and preoperative baseline test results of each group are summarized in Table 1.

**Table 1:** The demographics and baseline characteristic parameters of included patients and control individuals.

	Group A (n = 78)	Group P (n = 86)	Group C (n = 40)
Sex (F/M)	44/34	35/51	16/24
Age (year)	50 [44, 54]	49.5 [41, 54]	48 [39, 56]
Education level (year)	12 [9,12]	12 [9,16]	10.5 [9,13]
Height (cm)	162.58 ± 7.98	161.82 ± 7.69	162.05 ± 7.64
Weight (kg)	63.83 ± 10.22	60.53 ± 12.51	59.772 ± 9.9
BMI (kg/cm2)	24.06 ± 2.88	23.01 ± 3.86	22.70 ± 3.05
HR (bpm)	70 [65, 80]	74 [60, 75]	/
MAP (mmHg)	94.17 ± 13.55	90.88 ± 14.4	/
SPO2(%)	100 [98, 100]	99.26 [99, 100]	/
DST (pcs)	9 [8,10]	9 [7,10]	8 [7,10]
Schulte Grid(s)	19.7 [16.2, 23.7]	17.41 [14.2, 21.9]	18.8 [14.4, 25.6]
DSST (pcs)	6 [5,8]	7 [5,9]	6 [5,8]
Stroop(s)	41.0 [32.1, 55.8]	38.06 [31.8, 51.6]	41.3 [31.0, 54.3]
FTT-R(s)	8.6 [8.1, 9.4]	8.3 [7.5, 9.6]	8.5 [8.5, 9.3]
FTT-L(s)	9.2 [8.4, 10.1]	8.6 [7.8, 9.9]	8.9 [8.2, 10.1]

**Notes:** Data are presented as mean ± SD (normally distributed data), median [interquartile range] (skewed data).

**Abbreviations:**DST, digital span memory test; Schulte Grid, Schulte Grid test; DSST, digital symbol substitution test; Stroop, Stroop test; FTT-R, finger tapping test-right hand; FTT-L, finger tapping test-left hand; Group A, alfentanil group; Group P, propofol group; Group C, control group.

**Fig. 2:**
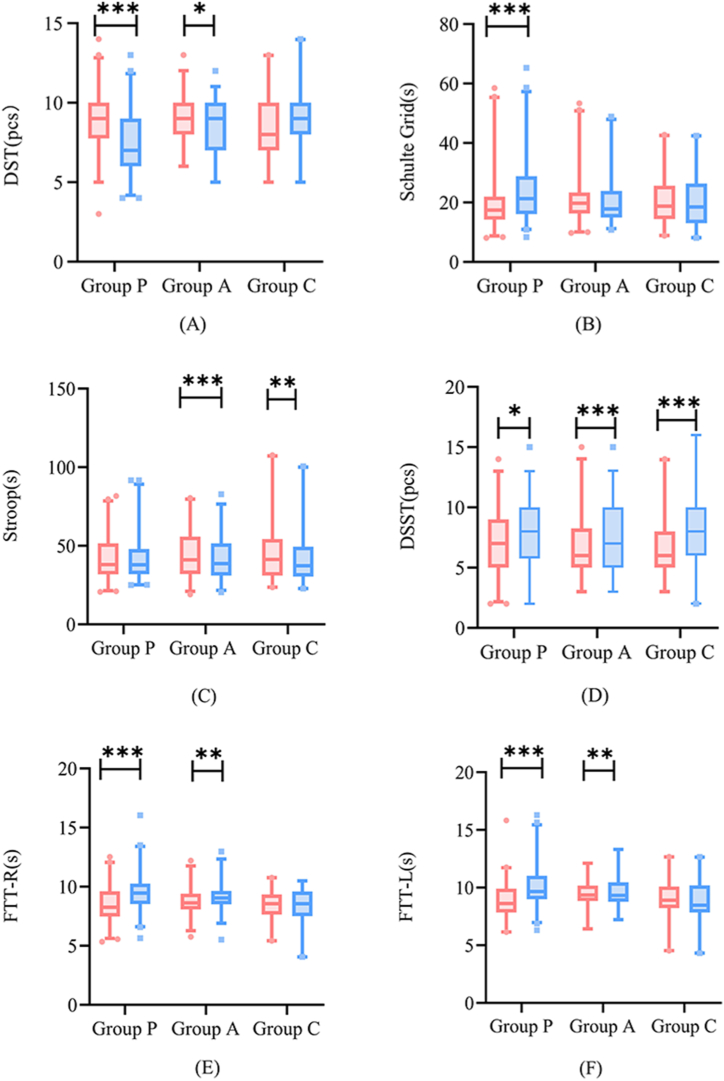
Distribution of various neuropsychological scores including DST (A), Schutle Grid (B), Stroop (C), DSST (D), FTT-R (E) and FTT-L (F) in patients of different groups and in the control group. Compared to scores before drug administration, *: P < 0.05 **: P < 0.01; ***: P < 0.001.

The distribution of the scores on various NPTs in the different groups is shown in Fig. 2. In the DST, group P showed a significant decrease in performance after the colonoscopy (P＜0.001), while the decrease from baseline in group A was statistically significant; there was no significant change in group C. In the Schulte table, only group P required a longer time to complete the task (P＜0.001), and there were no difference between groups A and C. In the DSST, the scores of groups A and C were significantly improved (P＜0.001), while group P showed an increase in performance. In the Stroop test, patients in groups A and C spent lesser time completing the test after the colonoscopy than before, while group P showed no difference. In addition, the time required to complete the task was longer in the FTT-R and FTT-L tests in both groups A and P, while the difference was not statistically significant in group C.

The results of the Z-score are shown in Table 2. The incidence of cognitive dysfunction at the discharge times was lower in group A than in group P (P < 0.001). The incidence of z scores >1.96 on percussion results was lower in group A than in group P (P < 0.001); The incidence of Z score >1.96 on the Schulte table was lower in group A than in group P (P = 0.03). The z scores >1.96 on digital memory tests were lower in group A than in group P (P = 0.01). In comparison, there was no significant difference in DSST (P = 0.32).

**Table 2:** Incidence of z score >1.96 on single neuropsychological test and incidence of cognitive dysfunction.

	Group A (n = 78)	Group P (n = 86)	P	RR (95%CI)
FTT-R (yes/no)	2/78 (2.5%)	17/86 (19%)	<0.001	0.13 (0.03–0.54)
FTT-L (yes/no)	5/78 (6.4%)	28/86 (33%)	<0.001	0.20 (0.08–0.49)
Schulte Grid (yes/no)	1/78 (1.2%)	12/86 (14%)	0.003	0.09 (0.01–0.69)
DST (yes/no)	2/78 (2.5%)	12/86 (14%)	0.009	0.18 (0.04–0.79)
DSST (yes/no)	7/78 (8.9%)	12/86 (14%)	0.320	0.64 (0.27–1.55)
Stroop (yes/no)	1/78 (1.2%)	5/86 (5.8%)	0.213	0.22 (0.03–1.85)
cognitive dysfunction (yes/no)	2/76 (2.5%)	20/86 (23%)	<0.001	0.11 (0.03–0.46)

**Notes:**Data are expressed as numbers (%) (classified data).

**Abbreviations:**DST, digital span memory test; Schulte Grid, Schulte Grid test; DSST, digital symbol substitution test; Stroop, Stroop test; FTT-R, finger tapping test-right hand; FTT-L, finger tapping test-left hand; Group A, alfentanil group; Group P, propofol group; Group C, control group.

The duration of anesthesia and surgery and the postoperative period and complications are recorded in Table 3. The operation time was 7 min in Group A and 9 min in Group P (P = 0.025). The discharge time of patients in groups A and P was also statistically different (P < 0.001) (Table 3). Postoperative pain scores were also lower in group A than in group P (P = 0.015), but the incidence of vomiting was higher in group A than in group P (P = 0.01). The satisfaction scores of the endoscopist and patients were similar between the two groups (Table 3).

**Table 3:** Sedation, procedure, recovery characteristics, and events.

	Group A (n = 78)	Group P (n = 86)	P
Anesthesia time (min)	8.5 [6,11]	12 [10,15]	＜0.001
Operation time (min)	7 [6,9]	9 [6,12]	0.025
Discharge time (min)	5 [3,9]	13 [10,18]	<0.001
OAA/S at 3min after operation	4 [4,4]	1 [1,1]	<0.001
Hypotension (yes/no)	3/75	19/67	0.001
Hypertension (yes/no)	2/76	3/83	0.900
Respiratory depression (yes/no)	1/77	0/86	0.290
Bradycardia (yes/no)	4/74	2/84	0.530
Postoperative nausea (yes/no)	8/70	1/85	0.010
Postoperative vomiting (yes/no)	0/78	0/86	1.000
Postoperative pain NRS	1 [0, 2]	1 [1,2]	0.015
Endoscopists satisfied score	5 [4,5]	5 [4.25, 5]	0.195
Patient satisfied score	5 [4,5]	5 [5,5]	0.379

**Notes:**Data are presented as mean ± SD (normally distributed data), median [interquartile range] (skewed data), or number (%) (categorical data).

**Abbreviations:** Group A, alfentanil group; Group P, propofol group.

**Fig. 3:**
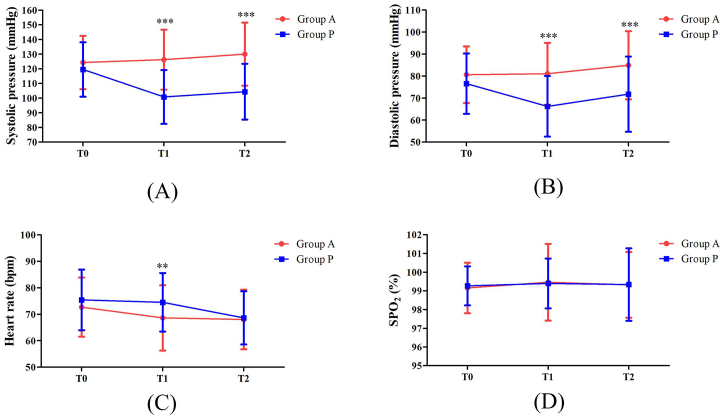
A: Systolic blood pressure at each time point in the alfentanil group and the propofol group; B: Diastolic pressure at each time point in the alfentanil group and the propofol group; C: Heart rate at each time point in the alfentanil group and the propofol group; D: SpO2 at each time point in the alfentanil group and the propofol group; T0 = baseline; T1 = at the beginning of operation; T2 = 3 min after operation; Group A = group of alfentanil, Group P = group of propofol, Group C = group of control; *: P < 0.05 **: P < 0.01; ***: P < 0.001.

Vital signs during anesthesia are recorded in Fig. 3. There was no significant difference in the heart rate and pulse oxygen saturation at T2 between the two groups. The incidence of bradycardia and respiratory depression in the operation center was similar between the two groups (Table 3). However, the incidence of hypotension in group A was significantly lower than that in group P (P < 0.001). In the two-way repeated analysis of variance of systolic and diastolic blood pressure; the decline in blood pressure in group P was also more significant than that in group A (Fig. 3).

## 4 Discussion

To the best of our knowledge, this study is the first to directly compare the postoperative cognitive functions of patients undergoing colonoscopy administered with alfentanil or propofol. The results showed that incidence of postoperative cognitive decline in patients receiving propofol was substantially higher than that in those receiving alfentanil.

In this study, we performed a second psychological test when the patient met the discharge criteria. In the FTT, we found that both groups experienced a significant decrease in postoperative performance from baseline, and the P group was even worse. The effect of propofol on the thalamus cortex and basal ganglion circuitry causes damage to the rhythm of finger movements [26], which can explain why propofol slows down finger movements. However, there is no current evidence to confirm the effects of alfentanil on the basal ganglia. A recent meta-analysis concluded that opioids do not affect patients' motor performance [27,28], this decrease might be due to decreased alertness and attention.

The scores of DST in group A decreased not significantly than that in group P，suggesting that alfentanil has a more negligible effect on memory than propofol. Animal experiments have shown that propofol may cause neuronal apoptosis and affect memory capacity [27]. Propofol is associated with memory processing, leading to memory impairment [28]. In healthy volunteer studies, memory capacity was not affected by alfentanil [29]; this is inconsistent with our findings. The decline in cognitive function may be related to intestinal preparation, hypotension, and hypoglycemia caused by fasting [30]. Additionally, the expression of locus coeruleus and μ-opiate receptors (MOR) can inhibit the activation of the locus coeruleus-norepinephrine system by stressors, thus affecting cognitive function [31]. while the effect on memory needs to be further studied.

In the Schult table and Stroop tests, only group P showed a significant drop in scores [32]. Therefore, we believe that the patients in group P were affected in terms of attention. Studies of the cerebral metabolic rate using positron emission tomography have shown that under propofol anesthesia, the metabolic rates of different brain regions are affected to different degrees [33]. The decrease in cerebral blood flow caused by propofol occurs in the frontal cortex, cuneus, precuneus, posterior cingulate gyrus, and retrosplenial cortex [34,35]. However, neuroimaging research suggested that attention and responsiveness are involved in these regions [36]. Stroop test scores are also related to the activation of brain regions, such as the anterior cingulate cortex, dorsolateral prefrontal cortex, and parietal lobe [37]. The Stroop test results in group A were similar to those in group C, and they achieved better results in the second measurement, which may be due to the learning effect.

Regarding the DSST, the score changes of group P were lower than those of group C and group A, but no significant difference in the z-score. Patients in group P were affected in cognitive areas of executive function. Propofol is known to act on GABA receptors, inducing the release of the inhibitory neurotransmitter GABA and producing anesthetic effects. Porphyuleus GABA neurons terminate in norepinephrine (NE) neurons, whose activation leads to reduced NE release and affects cognitive function [33].

Results from our NPTs showed that at discharge, the incidence of cognitive decline in group A was significantly lower than that in group P. Propofol leads to a more pronounced decline in fine motor function, attention, and other cognitive functions than afentanil. Additionally, more patients in group A return to work within 24 h, indicating that the longer time side effect on personal working ability of afentanil is also less than that of propofol. So that, alfentanil may provide a new reference for clinical medication.

In addition to NPTs, intraoperative vital signs were recorded and analyzed. The incidence of hypotension in group P was 22% during treatment, while alfentanil improved hemodynamic stability. The incidence of respiratory depression was low in both groups. Alfentanil inhibits breathing to a lesser extent than other opioids and has a lower incidence of choking [38]. Pre-oxygenation and slow bolus administration can reduce respiratory depression.

What's more, the operation time in group A was significantly shorter than that in group P. Those who only used alfentanil could better follow the doctor's advice during the procedure (such as changing the position and recalling the medical history) and cooperate more with the colonoscopy to some extent, accelerating the examination rhythm. Most patients met the discharge criteria after endoscopic procedures [12].

Nausea and vomiting are obvious side effects of opioids, and 40% of patients develop nausea after using opioids [39]. Fortunately, alfentanil causes less postoperative nausea and vomiting than equipotent doses of fentanyl or sufentanil in outpatients [40]. Visceral pain which caused by the activation of sensory nerves that dominate the intestinal tract, also triggers autonomic nervous responses, including nausea and vomiting [11]. In our study, the incidence of postoperative nausea in group A was still higher than in group P. For this reason, antiemetic drugs should be administered preoperatively to reduce the adverse reactions caused by opioids.

During the study, five patients were unable to tolerate colonoscopy and decided to add propofol for sedation to complete the colonoscopy under the evaluation of the anesthesiologist. It has been suggested that being male, a higher education level, lower preoperative anxiety, and personal preference for surgery without sedation can improve the success rate of colonoscopy without sedation. However, among the patients who successfully completed the colonoscopy, there was no overall significant difference in patient satisfaction scores between the two groups.

This study has some limitations. First, these results are currently only applicable to patients who are relatively healthy and have no serious illnesses. Considering that older patients (>65 years old) have less demand to return quickly to everyday work, they were not included in this experiment. Furthermore, we only measured the cognitive function level of patients at the time of discharge, and there was no answer as to when the patients could return to their preoperative status. In the future, it will be necessary to set more time points for cognitive evaluations.

## 5 Conclusion

In summary, colonoscopy performed with alfentanil alone resulted in lesser postoperative cognitive impairment and shorter hospital discharge time than those with propofol. In addition, the alfentanil group achieved similar patient and endoscopic physician satisfaction as that of the propofol group. In some patients who require rapid recovery, alfentanil alone can help complete the examination process and provide an option for current clinical medications.

## Author contribution statement

Xiwen Zhu: Xuehan Chen: Performed the experiments; Wrote the paper.

Xuemei Zheng: Performed the experiments.

Hongyao Lyu, Jie Chen: Ai Yan: Qi Liu: Analyzed and interpreted the data.

Shiqi Li, Yamei Zhang: Ting Wang: Contributed reagents, materials, analysis tools or data.

Guangyou Duan, He Huang: Conceived and designed the experiments.

## Data availability statement

Data will be made available on request.

## Additional information

No additional information is available for this paper.

## Funding

This work was supported by the Kuanren Talents’ Project of The Second Affiliated Hospital of 10.13039/501100004374Chongqing Medical University.

## Declaration of competing interest

The authors declare that they have no known competing financial interests or personal relationships that could have appeared to influence the work reported in this paper.

## References

[1] Bibbins-Domingo K. (2016). Screening for colorectal cancer: US preventive services task force recommendation statement. JAMA.

[2] Stewart M.J. (2022). Does surveillance increase survival? Benefits of periodic colonoscopy in patients with ulcerative colitis and colorectal cancer. Dig. Dis. Sci..

[3] Rutter M.D. (2016). The European society of gastrointestinal endoscopy quality improvement initiative: developing performance measures. United Eur. Gastroenter. J..

[4] Zhang X.L. (2013). Current application situation of gastrointestinal endoscopy in China. World J. Gastroenterol..

[5] Hirsh I. (2006).

[6] Zhou S. (2021). National survey on sedation for gastrointestinal endoscopy in 2758 Chinese hospitals. Br. J. Anaesth..

[7] Singh H. (2008). Propofol for sedation during colonoscopy. Cochrane Database Syst. Rev..

[8] Ko C.W. (2010). Serious complications within 30 days of screening and surveillance colonoscopy are uncommon. Clin. Gastroenterol. Hepatol..

[9] Sargin M., Uluer M.S., Şimşek B. (2019). The effect of bispectral index monitoring on cognitive performance following sedation for outpatient colonoscopy: a randomized controlled trial. Sao Paulo Med. J..

[10] Ekmekci P. (2017).

[11] Eberl S. (2012).

[12] Eberl S. (2014). Is "really conscious" sedation with solely an opioid an alternative to every day used sedation regimes for colonoscopies in a teaching hospital? Midazolam/fentanyl, propofol/alfentanil, or alfentanil only for colonoscopy: a randomized trial. Tech. Coloproctol..

[13] N'Kaoua B. (2002). Time course of cognitive recovery after propofol anaesthesia: a level of processing approach. J. Clin. Exp. Neuropsychol..

[14] Chung F., Chan V.W., Ong D. (1995). A post-anesthetic discharge scoring system for home readiness after ambulatory surgery. J. Clin. Anesth..

[15] Berger M. (2019). Flow cytometry characterization of cerebrospinal fluid monocytes in patients with postoperative cognitive dysfunction. A Pilot Study.

[16] Quan C. (2019). BIS-guided deep anesthesia decreases short-term postoperative cognitive dysfunction and peripheral inflammation in elderly patients undergoing abdominal surgery. Brain Behav..

[17] Deng Y. (2021). Methylene blue reduces incidence of early postoperative cognitive disorders in elderly patients undergoing major non-cardiac surgery: an open-label randomized controlled clinical trial. J. Clin. Anesth..

[18] Gualtieri C.T., Johnson, L.G.J.A.o.C. Neuropsychology (2006). Reliability and validity of a computerized neurocognitive test battery. CNS Vital Signs.

[19] Li M. (2015). The effect of Chinese traditional exercise-baduanjin on physical and psychological well-being of college students. Random. Contr. Trial.

[20] Kumpaitiene B. (2018). Correlation among decreased regional cerebral oxygen saturation, blood levels of brain injury biomarkers, and cognitive disorder. J. Int. Med. Res..

[21] Klinger R.Y. (2019). Intravenous lidocaine does not improve neurologic outcomes after cardiac surgery. Random. Contr. Trial.

[22] Türk H.e. (2013).

[23] Lee J.J. (2015). Pain Relief Scale Is More Highly Correlated with Numerical Rating Scale than with Visual Analogue Scale in Chronic Pain Patients.

[24] Silbert B. (2015).

[25] Zhang Y. (2018).

[26] Perika T. (2019). Psychomotor recovery of dexmedetomidine compared with propofol after sedation during spinal anesthesia. Random. Control Trial.

[27] Pearn M.L. (2012). Propofol neurotoxicity is mediated by p75 neurotrophin receptor activation. Anesthesiology.

[28] Quan X. (2013).

[29] Scamman F.L., Ghoneim M.M., Korttila K. (1984). Ventilatory and mental effects of alfentanil and fentanyl. Acta Anaesthesiol. Scand..

[30] Wadsworth P. (2015). Does bowel preparation for colonoscopy affect. Cognitive Function?.

[31] Guajardo H.M., Valentino R.J. (2021). Sex differences in μ-opioid regulation of coerulear-cortical transmission. Neurosci. Lett..

[32] Borrat X. (2019). Computerized tests to evaluate recovery of cognitive function after deep sedation with propofol and remifentanil for colonoscopy. J. Clin. Monit. Comput..

[33] Chen C. (2016).

[34] Fiset P. (1999).

[35] Veselis R.A. (2004).

[36] Fan J. (2003). Mapping the genetic variation of executive attention onto brain activity. Proc. Natl. Acad. Sci. U. S. A..

[37] Al-Zahrani M.A., Elsayed Y.A. (2009). The impacts of substance abuse and dependence on neuropsychological functions in a sample of patients from Saudi Arabia. Behav. Brain Funct..

[38] Schifftner C., Schulteis G., Wallace M.S. (2017). Effect of intravenous alfentanil on nonpainful thermally induced hyperalgesia in healthy volunteers. J. Clin. Pharmacol..

[39] Tamura T. (2021). Effect of prophylactic anti-emetics on opioid-induced nausea and vomiting: a retrospective observational cohort study. In Vivo.

[40] Langevin S. (1999).

